# A Novel Method for Culturing Telencephalic Neurons in Axolotls

**DOI:** 10.1002/cne.70066

**Published:** 2025-06-18

**Authors:** Sevginur Bostan, Safiye Serdengeçti, F. Kemal Bayat, Sadık Bay, AyşeServer Sezer, Neşe Ayşit, Gürkan Öztürk

**Affiliations:** ^1^ Research Institute for Health Sciences and Technologies (SABITA) Regenerative and Restorative Medicine Research Center (REMER) Istanbul Medipol University Istanbul Türkiye; ^2^ Department of Physiology Graduate School of Health Sciences Istanbul Medipol University Istanbul Türkiye; ^3^ Department of Physiology School of Medicine Istanbul Medipol University Istanbul Türkiye; ^4^ Department of Neuroscience Graduate School of Health Sciences Istanbul Medipol University Istanbul Türkiye; ^5^ Engineering Faculty Department of Electrical and Electronics Engineering Marmara University Istanbul Türkiye; ^6^ Chemical Biology Program Memorial Sloan Kettering Cancer Center New York New York USA; ^7^ Department of Medical Biology School of Medicine Istanbul Medipol University Istanbul Türkiye; ^8^ Department of Physiology, School of Medicine Abant Izzet Baysal University Bolu Türkiye

**Keywords:** axolotl, primary neuron culture, regeneration, telencephalon

## Abstract

The axolotl (*Ambystoma mexicanum*), a neotenic salamander with remarkable regenerative capabilities, serves as a key model for studying nervous system regeneration. Despite its potential, the cellular and molecular mechanisms underlying this regenerative capacity remain poorly understood, partly due to the lack of reliable in vitro models for axolotl neural cells. In this study, we developed a novel protocol for primary cultures of adult axolotl telencephalon/pallium, enabling the maintenance of viable and functionally active neural cells. Using calcium imaging and immunocytochemistry, we demonstrated the presence of neuronal and glial markers, synaptic connections, and spontaneous calcium activity, highlighting the functional integrity of the cultured cells. Our findings reveal that these cultures can be maintained in both serum and serum‐free conditions, with neurons exhibiting robust neurite outgrowth and responsiveness to injury. This protocol addresses a critical gap in axolotl research by providing a controlled in vitro system to study neurogenesis and regeneration. By offering insights into the regenerative mechanisms of axolotl neurons, this work lays the foundation for comparative studies with mammalian systems, potentially informing therapeutic strategies for neurodegenerative diseases and CNS injuries in humans.

## Graphical abstract

A protocol for primary neuron culture from axolotl telencephalon was developed. Spontaneous and axotomy‐induced calcium transients showed excitable cells and neuronal and glial characterization was performed with immunocytochemistry.

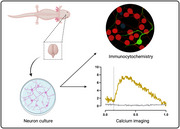

## 1 Introduction

The Mexican salamander, axolotl (*Ambystoma mexicanum*), an urodele amphibian that cannot complete metamorphosis and preserves neotenic characteristics throughout its lifespan, has been a focus of interest as a model organism in regeneration research (De Groef et al. 2018; Echeverri et al. 2022; Reiß 2022). Compared to rather limited regeneration ability of nervous system tissues in adult mammals, urodele amphibians have a much higher capacity to regenerate their nervous system after an injury (Amamoto et al. 2016; Lust et al. 2022; Maden et al. 2013; Tazaki et al. 2017).

Telencephalon region in the brain of these amphibians is known to contain a highly proliferative and regenerative ventricular zone (VZ) compared to other reptiles (Maden et al. 2013). There is very limited information on the structural and molecular mechanisms underlying this high regenerative capacity. The cellular organization and characteristics of different cell populations of the axolotl brain and how they functionally regenerate after injury have not been well defined. This is partially due to lack of reliable in vitro models to study regenerative processes under controlled conditions of cell culture. In general, in vitro studies using axolotl cell cultures are scarce and existing ones mostly focus on blastema‐derived cells (Ferretti and Kumar 2015). Only available axolotl cell line is AL‐1, whose origin is unclear (Roy et al. 2000). Primary cultures of neural cells are even less common; the only example is a protocol for generation of neurospheres from newt brain (Hameed and Simon 2015; Kirkham et al. 2014).

In this study, we developed a protocol for primary cultures of adult axolotl telencephalon/pallium. We tested the viability and maintenance of the primary culture with serum or serum‐free medium. We also carried out calcium imaging to demonstrate they are functionally active and responsive. In addition, we performed immunocytochemistry to determine cell types and synaptic connections in cultures.

## 2 Materials and Methods

### 2.1 Ethics Statement and Animal Handling

Axolotls were kept and bred at the Experimental Animal Centre of Istanbul Medipol University (MEDITAM). All animals were handled in strict accordance with guidelines for animal care and use issued by the EU directive code, 86/609/CEE. The Committee on Ethics of Animal Experimentation of Istanbul Medipol University (IMUHADYEK) approved all experimental procedures. Adult white axolotls (*n* = 6), 12–16 cm in length, were used in the optimization of the culture protocol and experiments.

### 2.2 Primary Telencephalon Culture Protocol

#### 2.2.1 Animal Surgery

**FIGURE 1:**
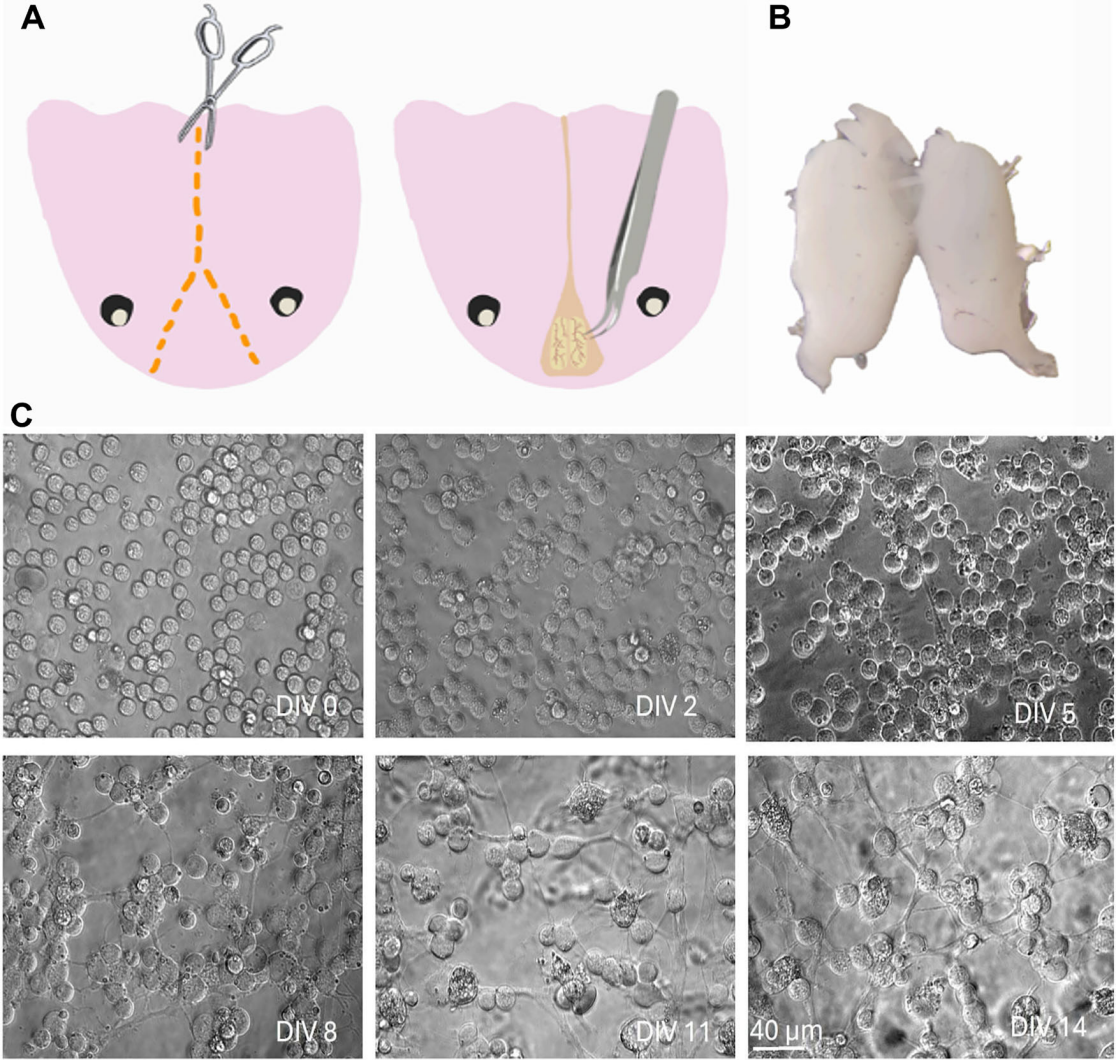
Axolotl primary telencephalon culture procedure. (A) Dissection and removal of the brain. (B) Isolated telencephalon. (C) Brightfield images of cultured neurons during 2 weeks of incubation.

Axolotls were anesthetized with 0.02% benzocaine (Sigma‐Aldrich, E1501) and decapitated swiftly. The skull was cut from the spinal cord to the median fontanel, following a straight line. Upon reaching the median fontanel, a triangular section was excised, as depicted in Figure 1A. The telencephalon was accessed at the site where the skull piece was removed, as indicated in Figure 1A. Using a forceps with curved tips, the telencephalon was carefully isolated and transferred into the dissection medium Leibovitz's L‐15 (L‐15‐Gibco) (1% Pen‐Strep [Sigma‐Aldrich] and 1% L‐glutamine [Sigma‐Aldrich]). Under a stereo microscope, the meninges were removed, and extracted tissue is shown in Figure 1B.

#### 2.2.2 Tissue Dissociation

Culturing protocol was based on an earlier study (Aydın et al. 2023) with several adjustments for axolotls. After dissection, both telencephalon hemispheres were transferred into a separate dissociation medium L‐15 (1% Pen‐Strep, 1% L‐glutamine) with Papain (12.5 U/mL, Sigma‐Aldrich) and incubated for 45 min at 4°C. Afterward, DNAse (50 µg/mL, BioMatik) was added, and the tissue pieces were gently triturated with fire‐polished glass Pasteur pipettes of decreasing diameters until a homogeneous cell suspension was obtained. Then, 10% FBS (Sigma‐F4135) is added to the cell suspension, and it is incubated for 15 min at room temperature (RT) for enzyme inhibition.

#### 2.2.3 Gradient Centrifugation

To obtain a neuron‐rich cell population free of tissue debris, a gradient centrifugation step was added, which was adapted from a previous study (Bektaş and Öztürk 2013). The cell suspension was carefully added on top of a four‐layer Percoll (Sigma) gradient which was prepared in L‐15 medium with 60%, 30%, 20%, and 10% densities. Then it was spun at 3000 × *g* for 25 min in a centrifuge at 15°C. The cells were collected from 30% layer, transferred into a fresh L‐15 medium (1% Pen–Strep, 1% L‐glutamine) and centrifuged for 5 min at 300 × *g* to remove Percoll.

#### 2.2.4 Cell Seeding

After discarding the supernatant, cells were resuspended with the culture medium Neurobasal‐A (NBA‐Gibco) (1% Pen–Strep, 1% L‐glutamine, 2% B‐27 (Gibco), with or without 2% horse serum (Sigma, H1270). Prior to seeding, polystyrene cloning cylinders (I.D. × H 4.7 mm × 8 mm, Sigma, C7983) were placed on 35 mm diameter dishes (WPI) precoated with PEI (0.05–0.1%, Sigma). Then, the cells were seeded into the cloning cylinders at three different confluencies: high confluency (HC) (12,000/140 µL), medium confluency (MC) (6000/140 µL), and low confluency (LC) (3000/140 µL). Culture plates were maintained in an incubator at 21°C with 2% CO_2_. Thereafter, 30–40 µL of fresh medium was added every 3–4 days. The cloning cylinders were removed on DIV14, and 1 mL of fresh culture medium was added to the plates, and half of the medium was replaced every 3–4 days. To test viability and observe the development of neurons in vitro, cultures were maintained in either culture medium with or without heat‐inactivated horse serum (2%, Gibco).

### 2.3 Essays for Viability and Neurite Outgrowth

The viability of cultured neurons was assessed using propidium iodide (PI, Invitrogen). We applied 7.5 µM PI and counted total cell number on brightfield images, while detecting PI+ dead cells with fluorescence, using the Zeiss Cell Observer Spinning Disk (SD) Microscope equipped with a 20*x*/0.30NA objective. Following the addition of PI, the culture medium was not replaced, and the cells were maintained under standard culture conditions until the subsequent imaging session. Three random regions of interest (ROIs) from all plates were imaged every 3 days until DIV14 (*n* = 4). For the analysis of viability, we subtracted the number of PI+ cells from total cell count and measured the ratio of number of living cells/number of total cells. To quantify neurite outgrowth, we captured brightfield images from multiple ROIs and measured the length of the longest neurites of each cell every 3 days until DIV14 with a light microscope (Zeiss). Depending on DIV, average 1–45 cells were measured for each time point (*n* = 4). All image analyses for viability and neurite outgrowth were carried out manually using ZEN software.

### 2.4 Axotomy

To elicit excitation in the cultured neurons to test their responsiveness to injury, in vitro axotomy was performed with a UV laser utility (Rapp Opto) integrated on Zeiss LSM880 confocal microscope. The protocol was adapted from Aydın et al. (2023). A brief pulse of laser light (337 nm or 355 nm), lasting 0.5–1 s, was directed at axons approximately 50–100 µm from the neuronal soma. The laser emitted approximately 1–30 pulses/s, each with a duration of 3 ns and an energy output of around 300 µJ. The laser was shot for 1–2 s until the cut was visible.

### 2.5 Calcium Imaging

To monitor spontaneous calcium transients in neurons, we used Calcium Green‐1 (AM) (Invitrogen, Molecular Probes) staining at DIV30. The medium was replaced with NBA without phenol red containing pluronic acid and Calcium Green‐1 (AM). Cells were incubated for 40 min at 21°C, then staining solution was replaced with culture medium. Zeiss Cell Observer SD Time‐Lapse Microscope equipped with a 20*x*/0.30NA objective was used for imaging. Recordings of 1‐ to 2‐min periods were taken for detecting spontaneous calcium activity. Calcium imaging combined with axotomy injury was carried out using Zeiss LSM880 confocal microscope as stated above. Calcium activity was observed for 1 min as baseline and for 1 min after axotomy with time‐lapse imaging.

### 2.6 Immunocytochemistry

Cell cultures were fixed on DIV21 in 4% paraformaldehyde (PFA, Sigma) for 15 min at RT. After PFA was removed, a blocking solution (0.1 mol/L PBS containing 3% bovine serum albumin [BSA], 0.3% sodium azide, 0.1% Triton X‐10) was added and incubated for 45 min at RT. After blocking, the preparation was incubated with primary antibodies in a dilution solution (0.1 mol/L PBS containing 3% BSA, 0.3% sodium azide, 1% Tween‐20) overnight at 4°C. Beta (β)‐III tubulin (tubulin 3/Tuj1) was localized using a rabbit polyclonal antibody used for detecting β‐III tubulin (Abcam, Cat#ab18207, RRID: AB_444319). Synaptophysin (SYP) was detected using a rabbit polyclonal antibody raised against amino acids 221–313 of SYP (Santa Cruz Biotechnology Cat#sc‐9116, RRID: AB_2199007). For detection of GFAP, we used recombinant monoclonal antibodies derived from mouse hybridoma (Andrews et al. 2019). Anti‐GFAP R416WT (N206B/9R) was a gift from James Trimmer (Addgene plasmid # 114536; RRID: Addgene_114536). The next day, the samples were washed with PBS and incubated with secondary antibodies (568 goat anti‐chicken, 488 goat anti‐mouse, and 488 goat anti‐rabbit (all from Invitrogen) for 3 h at RT. The preparations were imaged with a confocal microscope LSM800 (Zeiss). Quantification of β‐III tubulin+ and GFAP+ cells was performed by manual cell counting across 8 ROIs, with a total of 144 cells.

### 2.7 Statistical Analysis

All statistical analyses were done using GraphPad Prism 9. Serum and serum‐free groups were compared using multiple *t*‐test, significance values *p* < 0.05.

## 3 Results

First, as cloning cylinders were used to facilitate the better attachment of axolotl CNS cells, three different confluency ratios were tested for the area of the cloning cylinders. Cell viability was monitored for 14 days in vitro with PI labeling. There were no significant differences detected in terms of viability across serum and serum‐free conditions, except at DIV11 and DIV14 in the LC group (*p*‐value = 0.0229 and 0.0065, respectively). Among the three, medium level confluency was found to be the most suitable for other analysis. Cells seeded at other confluency conditions were either too crowded for neurite extension analysis (HC) or too sparse to create a neural network (LC).

**FIGURE 2:**
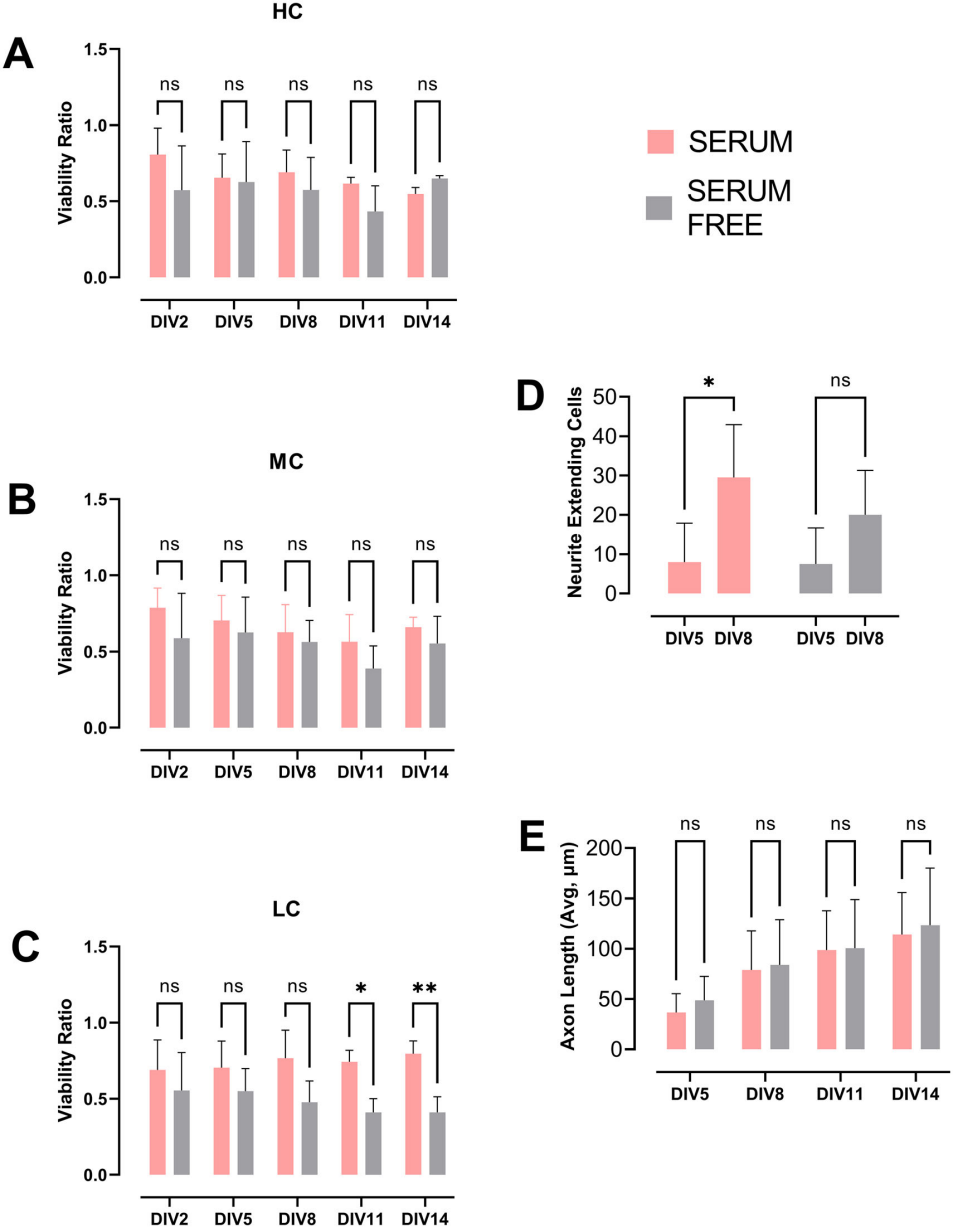
Viability and neurite extension in telencephalic cultures up to DIV14. (A–C) Normalized viability ratio of PI negative cells to total cell count in serum or serum‐free conditions in high (HC), medium (MC), and low confluency (LC) conditions, respectively. (D) Measurement of average maximum axon length over the course of 14 days in serum or serum‐free conditions. (E) Neurite extending cell count between DIV5 and DIV8 in serum (*p* value = 0.0414) and serum‐free (*p* value = 0.0707) conditions (*n* = 4).

The morphological development of the cells in vitro was tracked over a period of 14 days. Until the 5th day of culture (DIV5), cells were predominantly round with very little neurite outgrowth. Starting from DIV5, cells exhibited the initial stages of neurite growth (Figure 1C). Cells began to establish connections with neighboring cells, and a network started to form across the plate by DIV8–11 (Figure 1C). By DIV14, a dense and interconnected network of neurites had formed throughout the culture. The number of neurite‐bearing cells in the serum group exhibited a significant increase between DIV5 and DIV8 (*p*‐value = 0.0414); a similar tendency was observed in the serum‐free group, albeit not significant (*p*‐value = 0.0707) (Figure 2D). Axon lengths were measured every 3 days between DIV5 and DIV14. No significant difference in axon length was observed between the serum and serum‐free groups during this period (Figure 2E).

**FIGURE 3:**
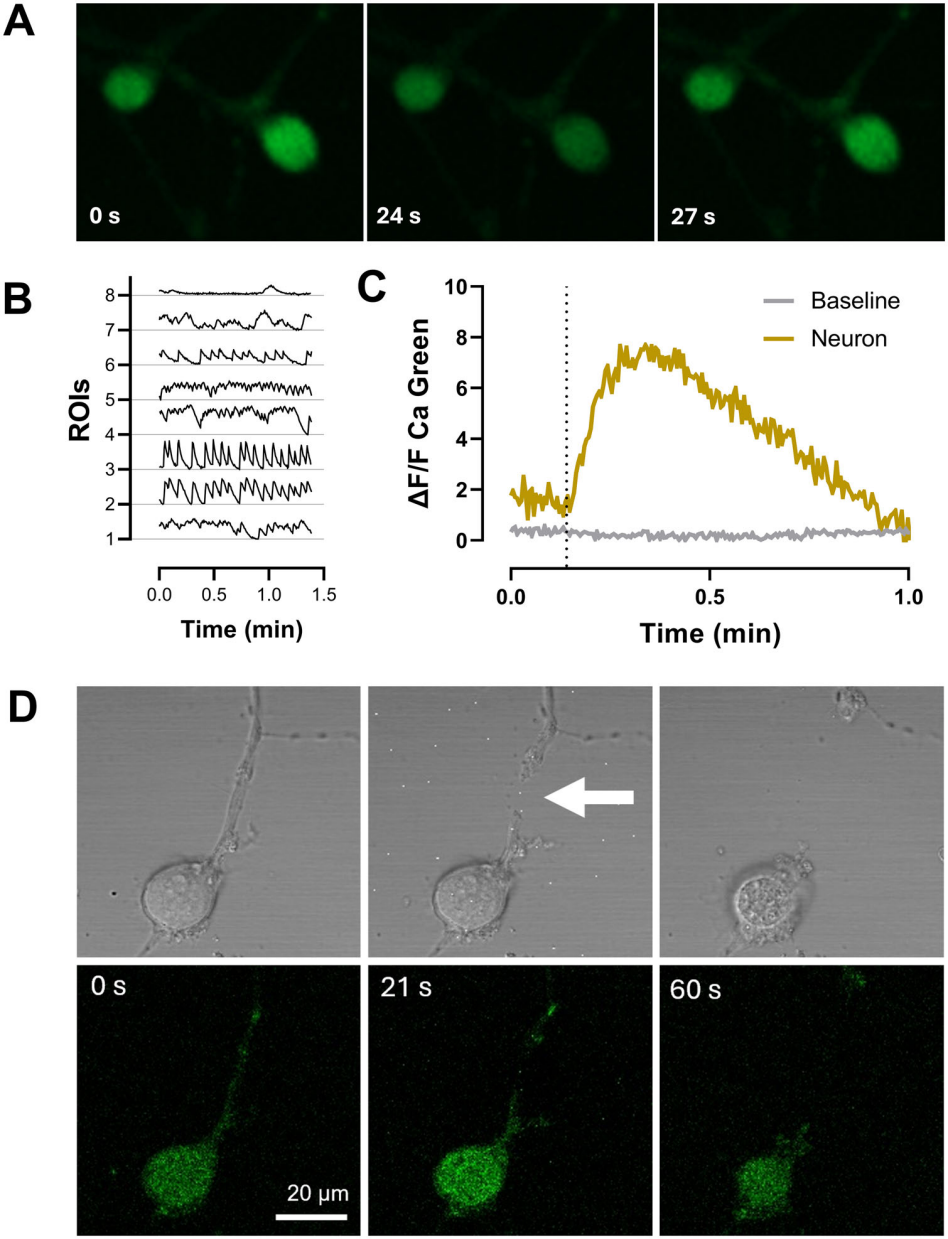
Calcium imaging and axotomy injury. (A) Representative images of spontaneous calcium activity detected with Calcium Green‐1 (AM). (B) Representative fluorescence traces of calcium activity from 8 ROIs. (C) Time series representation of calcium activity in panel D, axotomy time point indicated with dashed line. (D) Representative images showing calcium imaging of an axotomized neuron, arrowhead points to the injury site.

Spontaneous calcium activity was recorded over a 90‐s period using Calcium Green‐1 (AM) (Figure 3A). The observed spontaneous calcium activity indicates that these cultures contain active neurons with different firing frequencies. The number of spontaneous calcium events varied between 1 and 14/min as seen in Figure 3B. We also performed a laser axotomy injury model during calcium imaging to test the responsiveness of the cultures to injury. As seen in Figure 3C,D, axotomized neuron showed significant increase in intracellular calcium level and activity after axotomy.

**FIGURE 4:**
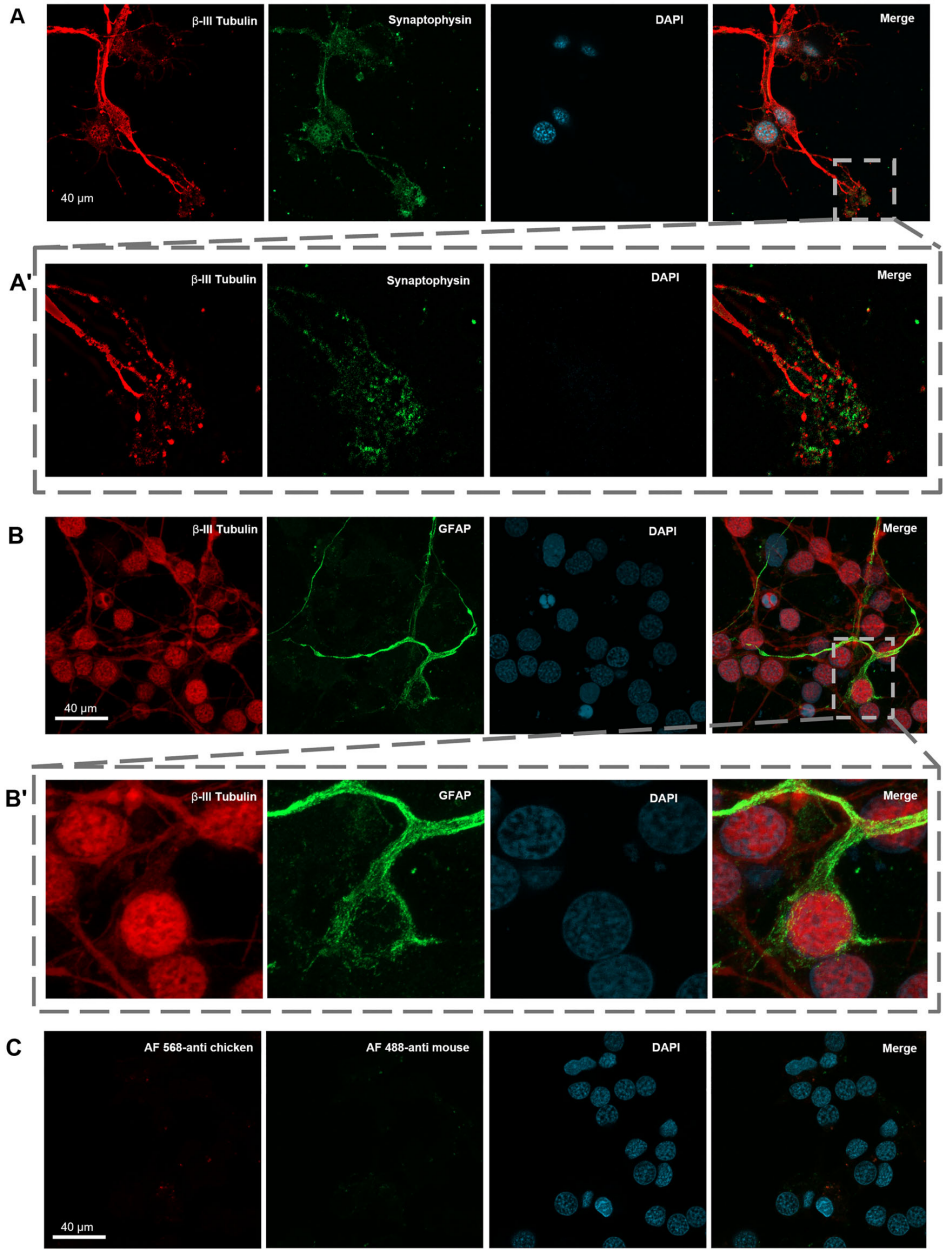
Representative ICC images of axolotl primary telencephalon culture. (A) Cells expressing β‐III tubulin (red), synaptophysin (green), and DAPI (cyan), indicating a neuron identity (scale bar = 40 µm). (A’) Inlet image from selected rectangular area showing axons and filopodia region with positive immunoreactivity for β‐III tubulin (red) and synaptophysin (green). (B) Cells stained for β‐III tubulin (red), GFAP (green), and DAPI (cyan) (scale bar = 40 µm). (B’) Inlet image from selected rectangular area showing one cell with immunoreactivity for GFAP (green) in the cytoplasm and for β‐III tubulin (red) in both cytoplasm and the nucleus. (C) Secondary antibody control for panel B images.

On DIV21, we performed immunocytochemistry to identify cell types and morphology of neural network formation. First, β‐III tubulin and SYP labeling indicated these neurons are capable of making synaptic connections (Figure 4A,A'). While the majority of the cells were identified as β‐III tubulin+ neurons both morphologically and with neural markers, we also observed some cells expressing both β‐III tubulin and GFAP. Around 7% of neurons showed coexpression of both GFAP and β‐III tubulin, suggesting a subset of cells may exhibit overlapping glial and neuronal characteristics. Moreover, we observed β‐III tubulin immunoreactivity in the nuclei of almost all cell types, which we think was a nonspecific cross‐reactivity (Figure 4B,B') as secondary antibody controls showed no such signal (Figure 4C).

## 4 Discussion

Primary cultures are indispensable to study nerve cells in controlled environment and widely used to investigate physiological and pathological processes of the nervous system. Regeneration of nervous tissue after a damage is an important topic for such studies and often refers to axonal regrowth and less frequently to neurogenesis. Although axolotl is a highly successful organism in both processes there is a lack of an established culture protocol specifically for axolotl nervous system cells. In this study, we successfully developed a method for culturing primary telencephalic cells from the axolotl brain, which yielded both neuron and glial marker positive cells as well as forming a neural network in time.

Several studies demonstrated different methods of culturing various tissues of axolotls; however, there has not been any established cell lines except for the AL‐1 line yet. As one of the main focuses of axolotl studies is investigating regeneration, blastema tissue emerging in early stages of limb regeneration is widely studied both in vivo and in vitro.

There are numerous studies for culturing the blastema tissue (Albert and Boilly 1988; Lehrberg and Gardiner 2015; Aladağ et al. 2024). There is also one study that demonstrates a protocol for culturing neurospheres from newt brains. They showed neural stem cells (NSCs) isolated from newt telencephalon formed neurospheres in culture expressing neuronal stem cell markers (Hameed and Simon 2015). Moreover, after maintenance with differentiation medium with growth factors for 14 days, Tuj1+ neurite extending and GFAP+ cells were detected in these neurospheres (Hameed and Simon 2015; Kirkham et al. 2014). Even though these findings substantiate the highly regenerative capacity of the telencephalic region of amphibian brains, they focus on the presence of stem cell markers and formation of neurospheres in cultures derived from newt brains.

We based the protocol for this axolotl telencephalon culture on our previous primary neuron culture protocol in mice (Aydın et al. 2023) with several adjustments for axolotl cells. In previous examples of amphibian and axolotl primary culture protocols, the temperature and CO_2_ levels for cell maintenance were set more suitable to amphibian physiology. As they are cold‐blooded animals, cultured cells are better maintained at temperatures between 20°C and 25°C. Also, CO_2_ levels at 2% were shown to balance pH levels more suitable for these cells (Ferretti and Kumar 2015). We used L‐15 medium for dissection and culturing steps as this medium supports cells in environments without CO_2_ equilibration. We also preferred using NBA medium for culture medium upon seeding the cells as it is designed for long‐term maintenance of postnatal and adult brain neurons when supplemented with B‐27 (Brewer 1995; Brewer et al. 1993). We wanted to test if additional horse serum will make an impact on cell viability. Though, horse serum is known to have a neuroprotective effect on primary neuronal cultures (Uto et al. 1994; Dux et al. 1996); our findings demonstrate that these cultures can be maintained either in serum or serum‐free conditions with slight differences in viability as shown in our results (Figure 2A–E). In limb regeneration studies in axolotls and newts, the complex crosstalk between nerve and blastema tissues was found to involve various growth factors playing a role (Gómez and Echeverri 2021). Considering aforementioned studies and our results, this finding indicates there is still need for further research on how growth factors influence the regeneration mechanisms in this species. The cloning cylinders we used in the first 2 weeks upon seeding are commonly used in isolating cell populations in certain culture conditions. However, we observed the conserved area they provide in glass bottom plates also facilitate the attachment of axolotl neurons without clumping when used in combination with PEI coating (Figure 1C), which is usually a challenge in amphibian primary cultures.

We added a centrifuge gradient step to the culture protocol to isolate neuron population from the telencephalon tissue; however, we observed a coexpression of neuron and glia markers in our cultures, with some cells showing both β‐III tubulin and GFAP immunoreactivity. One limitation of immunocytochemistry in axolotls is that there is a lack of reactive antibodies specifically manufactured targeting this species. Therefore, the expression of β‐III tubulin in the nucleus most likely is an artifact but may also indicate differentiating progenitor cells. Secondary antibody controls for Figure 4B showed no such signal in the nucleus (Figure 4C). In homeostatic conditions, ependymoglia proliferation is observed along the entire VZ in axolotls and increases in injury state (Lust and Tanaka 2019). In a recent study, Lust et al. (2022) found out that axolotl ependymoglia act as long‐term stem cells during homeostatic neurogenesis and can go through a transcriptional change that is specific to injury state. During the dissection and dissociation of neural tissue, the enzymatic and mechanical dissociation in obtaining cultures can also be considered a type of injury to the network of neurons as the neurites get severed (Kaplan et al. 2014). This may be a reason to see increased transcriptional change and, hence, expression of both ependymoglial and neuronal markers simultaneously in our cultures. However, further studies monitoring this expression at longer periods in vitro is needed. Our method also allows us to model and analyze the neurite outgrowth both at a single neuron and a network level. Neurite outgrowth is an essential feature of neurons in development and also in the adult brain, which makes it an important parameter that is used to monitor and analyze neuronal health and axonal regeneration in in vitro studies (Billnitzer et al. 2013; Khodosevich and Monyer 2010). Longest neurite outgrowth analysis showed neurons in these cultures can extend over 100 µm average distance in the course of 2 weeks maintaining the capacity of neuritogenesis and regeneration, which is similar to examples from rat and mice primary neuron cultures in the previously cited studies.

Moreover, the spontaneous calcium activity recorded in our cultures suggests that these neurons retain functional properties, while also showing response to injury induced by laser ablation. This functional aspect is critical for understanding the dynamics of neuronal signaling during regeneration and could provide insights into how axolotls manage to re‐establish neuronal networks after injury (Amamoto et al. 2016).

Limb regeneration in axolotls is also known to be nerve‐dependent both in the initial stages and maintenance (Kumar et al. 2007; Farkas et al. 2016; Wells et al. 2021), which makes it crucial to have coculture models or systems to investigate molecular mechanisms that drive these processes more deeply. This optimized culture protocol provides a valuable tool for future studies aimed at elucidating the regenerative capacity of axolotl CNS neurons and for conducting comparative studies with mammalian neurons to understand the key differences that contribute to their superior regenerative abilities. Furthermore, our findings align with the growing body of literature that emphasizes the importance of comparative studies between axolotls and mammals. By understanding the molecular and cellular differences that underpin the regenerative abilities of axolotls, researchers can identify potential targets for enhancing regeneration in less capable species (Vieira et al. 2020). The insights gained from our axolotl neuronal cultures could thus contribute to broader regenerative biology research, potentially informing approaches to treat neurodegenerative diseases or injuries in humans.

In summary, the development of this culture protocol is the first example of axolotl primary telencephalon culture, and it serves as a platform for exploring the fundamental principles of neurogenesis and regeneration in axolotls. Future studies using this method and also comparisons across species will contribute to unraveling the complex mechanisms that enable axolotls to regenerate their CNS, with implications for regenerative medicine and therapeutic interventions in humans.

## Author Contributions

S.Ba., N.A., and G.Ö. conceptualized the study and secured resources. S.Bo., S.S., F.K.B., and S.Ba. designed and performed the experiments, conducted data analysis, and interpreted results. S.Bo., S.S., F.K.B., and A.S.S. wrote the initial manuscript draft and prepared figures. G.Ö. critically reviewed and edited the manuscript and supervised the project. All authors reviewed and approved the final manuscript.

## Conflicts of Interest

The authors declare no conflicts of interest.

## Peer Review

The peer review history for this article is available at https://publons.com/publon/10.1002/cne.70066.

## Data Availability

All relevant data and resources can be found within the article and its Supporting Information.
